# Functional and Phenotypic Changes of Natural Killer Cells in Whole Blood during *Mycobacterium tuberculosis* Infection and Disease

**DOI:** 10.3389/fimmu.2018.00257

**Published:** 2018-02-19

**Authors:** Mathieu Garand, Martin Goodier, Olumuyiwa Owolabi, Simon Donkor, Beate Kampmann, Jayne S. Sutherland

**Affiliations:** ^1^Vaccines and Immunity Theme, Medical Research Council Unit, Fajara, Gambia; ^2^London School of Hygiene and Tropical Medicine, Bloomsbury, London, United Kingdom

**Keywords:** interferon gamma, CD107a, flow cytometry, CD57, NKG2C, Fc gamma receptor IIIa, innate memory, natural killer cells

## Abstract

Tuberculosis (TB) is still a global health concern, especially in resource-poor countries such as The Gambia. Defining protective immunity to TB is challenging: its pathogenesis is complex and involves several cellular components of the immune system. Recent works in vaccine development suggest important roles of the innate immunity in natural protection to TB, including natural killer (NK) cells. NK cells mediate cellular cytotoxicity and cytokine signaling in response to *Mycobacterium tuberculosis* (Mtb). NK cells can display specific memory-type markers to previous antigen exposure; thus, bridging innate and adaptive immunity. However, major knowledge gaps exist on the contribution of NK cells in protection against Mtb infection or TB. We performed a cross-sectional assessment of NK cells phenotype and function in four distinct groups of individuals: TB cases pre-treatment (*n* = 20) and post-treatment (*n* = 19), and household contacts with positive (*n* = 9) or negative (*n* = 18) tuberculin skin test (TST). While NK cells frequencies were similar between all groups, significant decreases in interferon-γ expression and degranulation were observed in NK cells from TB cases pre-treatment compared to post-treatment. Conversely, CD57 expression, a marker of advanced NK cells differentiation, was significantly lower in cases post-treatment compared to pre-treatment. Finally, NKG2C, an activation and imprinted-NK memory marker, was significantly increased in TST+ (latently infected) compared to TB cases pre-treatment and TST− (uninfected) individuals. The results of this study provide valuable insights into the role of NK cells in Mtb infection and TB disease, demonstrating potential markers for distinguishing between infection states and monitoring of TB treatment response.

## Introduction

With an estimated 10.4 million new tuberculosis (TB) cases and 1.8 million deaths reported in 2015, TB is the leading cause of death from an infectious disease worldwide (1). The prevalence of *Mycobacterium tuberculosis* (Mtb) infection in Africa is among the highest in the world. Although TB treatments have successfully averted approximately 49 million deaths globally in the last decades, important gaps still exist in combating the epidemic. For example, there are currently no vaccines against any forms of adult TB (2) and no reliable biomarkers to distinguish latent from active TB status and, importantly, to determine the risk of developing the disease (3, 4). Advancing the understanding of TB immunobiology, particularly with regard to innate cells, is critical in developing novel interventions to combat TB.

At the site of the infection, interactions between Mtb and antigen-presenting cells, such as alveolar macrophages and dendritic cells, are the initial step of the anti-Mtb responses and lead to the presentation of Mtb antigens to CD4^+^ and CD8^+^ T cell in the lymph nodes. While the role of B cells can be ambivalent, B cells are also known to present Mtb antigens, secrete cytokine, and generate Mtb-specific antibodies; each of these events can influence the immunological milieu in favor of diverse adaptive immune responses, such as Th1, Th2, or Th17. Several immune mechanisms, involving CD4^+^, CD8^+^, and γδ T cells, have been shown to contribute to the control of Mtb after an infection has been established (5–8). The most important feature of the adaptive immune response to TB is associated with CD4^+^ T cells production of interferon gamma (IFNγ), a critical factor for protection against the disease (9), and have been the subject of substantial research [reviewed in Ref (10)]. The overall T cell adaptive responses during TB are reviewed elsewhere (3, 11).

In humans, however, the adaptive response to Mtb (measured by a positive reaction to a tuberculin skin test (TST) or interferon gamma release assay) is characteristically delayed compared with other infections. Therefore, engagement and activation of innate cells at the infection site is a major form of protection against TB (12). In addition to macrophages and dendritic cells, other innate cells, such as neutrophils and natural killer (NK) cells, also participate in the response to Mtb infection. NK cells are potent producers of IFNγ and provide signals to infected dendritic cells and macrophages to assist with mycobacteria elimination (13–16) [also recently reviewed in Ref. (17)]. Neutrophils have been shown to interact with NK cells and promote “licensing” of NK cells (i.e., the activation of a necessary inhibitory receptor on NK cells) (18). Interestingly, depletion of neutrophils has been reported to affect NK cell maturation, functions (19), and activation (20). These features of neutrophils highlight the importance of using whole blood in innate cell response assays since neutrophils are generally removed during peripheral blood mononuclear cells preparation. Appreciation of the role of NK cells during TB has only recently re-emerged and mounting evidence suggests that cell-mediated innate immunity against TB is a promising new tool against TB (17).

Natural killer cells mediate cellular cytotoxicity and cytokine signaling in response to antigens and are important mediators of innate immunity. In addition, some NK cells display specific memory-type markers to previous antigen exposures, forming a bridge between the innate and adaptive immune systems (17). Various subsets of NK cells have been described, and each possesses different levels of cell-mediated cytotoxicity and cytokine production (21). Portevin et al. showed that NK cells expressing different killer-cell immunoglobulin-like receptors haplotypes respond to varying degree to Mtb (22). In humans, infections with cytomegalovirus (CMV) (23–25), hepatitis B and C virus (26), hantavirus (27), and chikungunya virus (CHIKV) (28) lead to imprinted NK cell receptor repertoires with increased frequencies of specific NK cell subsets. Interestingly, in CMV infection, NKG2C^+^ NK cells are elevated during the acute phase of the disease, at which the level then persists up to a year post infection (29, 30). In response to CHIKV infection, the repertoire of NK cell receptors (activating and inhibitory) has been shown to be modulated; the increased in NKG2C^+^ NK cells with infection correlates with viral load (28). In addition, NKG2C genotype could be associated with disease state (31, 32) and may affect the frequency of NKG2C^+^ NK cells in adults (33). For example, deletion of the KLRC2 gene, which encodes NKG2C, is associated with psoriasis (34) and may have a role in the control of HCMV in children (35) but not in bacterial infection caused by *Chlamydia trachomatis* (32). Thus, the NKG2C genotype may have putative functional implication for NK cell activation (36). Interestingly, KLRC2 deletion frequency has been shown to vary substantially between populations, but the reason for this variation remain unknown (32). Although the task of defining the physiological role of each NK cell subset is nontrivial, such functional characterization could provide the critical knowledge for the development of NK cell-based directed therapy against malignancies and infectious diseases.

Evidence for the role of NK cells in the immune response to Mtb infection are sparse, especially when we look for human cohort from TB-endemic countries, comparison between TB patients pre- and post-treatment and latent TB infection, and/or analysis of fresh whole blood samples. In TB, NK cells populate the granuloma and can help control the infection via different mechanisms: (1) directly *via* cytotoxic activity (e.g., release of perforin and granulysin) (37–39), (2) indirectly *via* signaling to the adaptive system (3, 4, 40–42) and possibly *via* “innate memory” features (17) such as the specific expansion of the NKG2C^+^ subset as discussed above in other disease models. Nevertheless, knowledge about specific NK cell subset distribution (e.g., surface expression levels of Fc receptor, differentiation marker, and memory-like marker) and function in relation to TB and Mtb infection status in patients are limited (17, 43). This knowledge gap impedes the effort in defining protective immunity against TB and, consequently, our understanding of TB pathogenesis.

We hypothesized that NK cell function and subsets in the blood can inform both Mtb infection status and the TB treatment response. The objective of this cross-sectional study was to provide a description of NK cell function and subset frequencies in response to antigenic stimulation, including Mtb-specific antigens, in TB patients and their exposed household contacts (either presumed-infected, TST+, or uninfected, TST−). Investigation of NK cell receptor expression, differentiation phenotype, and functional characteristics in this study provides further insight into the role of NK cells in innate immunity to TB.

## Materials and Methods

### Patient Recruitment and Blood Sampling

This study was nested within the Medical Research Council (MRC) Unit The Gambia (MRCG) Tuberculosis Case Contact (TBCC) platform (44) and was approved by the MRCG and the Gambian Government-Joint Ethics Committee. Following written informed consent obtained from patients or parents/guardians, active smear-positive TB cases (R-cases) and their household contacts were recruited. A separate group of individuals, who were previously diagnosed with active TB and recruited by TBCC, were also included in this study when they visited the clinic for their 6-month post TB treatment follow-up (6mo-cases). Individuals with diagnosed active TB disease received the standard anti-TB therapy consisting of a 2-month intensive phase (daily isoniazid, rifampicin, ethambutol, and pyrazinamide) and a 4-month consolidated phase (daily isoniazid and rifampicin). Household contacts were further divided based on their TST results as TST+ or TST−. Table 1 summarizes the participant characteristics for each group. TST was carried out with 2 tuberculin units purified protein derivative (PPD, RT23, Statens Serum Institute, Copenhagen, Denmark) using the Mantoux method. Indurations were recorded at 48–72 h; a subject was positive for TST if the induration diameter was ≥10 mm. The study participants were ≥16 years old and approximately 10 mL of heparinized blood was collected from each patient. Absolute count for granulocytes and lymphocytes was obtained from whole blood prior to stimulation using the Medonic M-series M32 Hematology Analyzer (Boule Medical AB, Sweden).

**Table 1:** Summary of participant characteristics.

	R-cases (*n* = 20)	6mo-cases (*n* = 19)^a^	TST+ (*n* = 9)	TST− (*n* = 18)
Median age, years (range)	31.5 (17–65)	30 (18–69)	42 (20–65)	22.5 (16–51)
Female sex, *n* (%)	5 (25)	6 (23)	8 (89)	14 (78)
FflV + , *n* (%)	1 (5)	1 (5)	0	0

**Smear grade**^b^				
3, *n* (%)	7 (35)	4 (21.1)	N/A	N/A
2, *n* (%)	6 (30)	6 (31.6)	N/A	N/A
l, *n* (%)	6 (30)	7 (36.8)	N/A	N/A
neg, *n* (%)	1 (5)	2 (10.5)	N/A	N/A

**Chest X-ray grade**^c^				
3, *n* (%)	14 (70)	14 (73.7)	N/A	N/A
2, *n* (%)	5 (25)	5 (26.3)	N/A	N/A
l, *n* TO	1 (5)	0	N/A	N/A

^a^A separate group of individuals, who were previously diagnosed with active TB and recruited in the TBCC, were also included in this study when they visited the clinic for their 6-month post TB treatment follow-up (6mo-cases); i.e., the diagnostic assessments (referred to in this table) were performed 6 month prior to their inclusion in this study. Refer to the *Patient Recruitment and Blood Sampling* in the Section “Materials and Methods.”

*^b^Smear grade during diagnostic assessment: neg = no disease, 1 = minimal disease. 2 = moderate disease, 3 = advanced disease*.

*^c^Chest X-ray grade during diagnostic assessment: 1 = minimal disease, 2 = moderately advanced, 3 = far advanced*.

### Cell Stimulation

The blood cell stimulation protocol is based on previously published methods (45, 46). Heparinized whole blood from the patient was mixed gently with equal ratio of Roswell Park Memorial Institute medium (RPMI, Sigma, UK) and 175 µL of the mixture was stimulated with phytohemagglutinin (PHA; Sigma, UK) (5 µg/mL), recombinant IL-12 (PeproTech, USA) (5 ng/mL) and recombinant IL-18 (Medical and Biological Laboratories Co Ltd., Japan) (50 ng/mL) (this combination is referred to High Concentration Cytokines, HCC), PPD (Staten Institute, Denmark, Lot:#231) (10 µg/mL), early secretory antigenic target 6 culture filtrate protein 10 fusion protein (ESAT-6/CFP10, EC); kindly provided by Prof. Tom H. M. Ottenhoff, Leiden University Medical Center, or RPMI. All conditions contained low concentration of rIL-12 (12.5 pg/mL) and rIL-18 (10 ng/mL), except for HCC. CD107a was added to each well (to a final concentration of 0.2%) and the plate incubated for 15–16 h at 37°C, 5% CO_2_. The concentrations of PPD and EC, as well as the duration of stimulation, have been previously validated in our laboratory and by other groups (47–53). Subsequently, 1X protein transport inhibitor (eBioscience, UK) was added and incubation continued for a further 3 hrs. EDTA was then added (final concentration, 2 mM) and the strip incubated for another 15 min at 37°C. The content from each well was then transferred to pre-filled cryogenic tubes containing FACSLyse buffer (1.2 mL; Becton Dickinson, USA), inverted to mix gently, and stored immediately at −80°C until staining.

### Intracellular Cytokine Staining and Flow Cytometric Acquisition

Sample preparation for flow cytometry was performed as previously described (45, 46, 54). Briefly, frozen tubes were thawed and spun (600 x *g* for 5 min). Pellets were then suspended in 200 µL of BD FACS Permeabilizing Solution 2 (Becton Dickinson, USA) and incubated for 10 min in the dark. After two washes with FACS buffer (PBS supplemented with 5% BSA, 10% NaN_3_ (all from Sigma, UK), and adjusted to pH 7.4–7.6), samples were labeled in a final volume of 50 µL with a cocktail of fluorochrome-labeled antibodies including: CD57 FITC (Cat# 555619 Lot# RRID:AB_395986), CD16 PE-CF594 (Cat# 562293 Lot# RRID:AB_11151916), CD14 PE-Cy7 (Cat# 557742 Lot# RRID:AB_396848), CD3 V450 (Cat# 560365 Lot# RRID:AB_1645570), CD45 V500 (Cat# 560777 Lot# RRID:AB_1937324), CD56 APC (Cat# 555518 Lot# RRID:AB_398601), IFNγ Alexafluor700 (Cat# 557995 Lot# RRID:AB_396977), CD8 APC-H7 (Cat# 560179 Lot# RRID:AB_1645481) (all from BD Biosciences, UK), and NKG2C/CD159c PE (R and D Systems USA, Cat# FAB138P Lot# RRID:AB_2132983), CD107a BV605 (BioLegend USA, Cat# 328634 Lot# RRID:AB_2563851). After staining, samples were washed with FACS buffer and immediately acquired on a BD Fortessa (Becton Dickinson, USA). Compensation Beads (CompBeads; BD Biosciences, UK) were used to standardize the voltage settings and used as the single-stain positive and negative controls. A total of 400,000 uncompensated events were acquired from each sample and compensation was set in FlowJo V.10 (FlowJo Tree Star USA, RRID:SCR_008520). Gating during analysis was based on the fluorescence-minus-one principle (55, 56). Cell viability was assessed *via* Forward and Side Scatter (FSC/SSC) appearance as previously described (45, 54). The minimum number of gated cell events accepted for analysis was 100 events.

### Statistical Analyses

GraphPad Prism v.5 (Graphpad Prism Software USA, RRID:SCR_002798) was used for all plots and statistical analyses. For certain cell subsets, the robust regression and outlier removal (ROUT) method was used to identify outliers within stacks of values (i.e., participant groups) (57). The Kruskal–Wallis non-parametric test, followed by Dunn’s *post hoc* test, was used to identify significant differences between groups for each cell subset. The effect of stimulation within a subject group was analyzed using Freidman test for repeated measured with Dunn’s *post hoc* test and comparisons were performed against the unstimulated condition. The effects of age, HIV, smear grade, and chest x-ray grade on cell subset frequencies was assessed using generalized linear model (glmnet package in R, RRID:SCR_015505) (58). These parameters had no effects on the classification model, thus, all results are presented as unadjusted values.

## Results

### Participant Characteristics

Table 1 summarizes the participant characteristics. A total of 66 subjects were included in the study. The median ages for each group were comparable except for the TST− group which was lower compared to the other groups (non-significant). Participants in the R-cases and 6mo-cases groups were majorly male while those of TST+ and TST− groups were majorly female. Two (3%) of the study participants, belonging to R-cases and 6mo-cases, were HIV positive. The distribution of smear grades and chest x-ray scores, determined at recruitment into TBCC, was similar for individuals in the R-cases and 6mo-cases groups.

### Phenotypic Assessments

**Figure 1:**
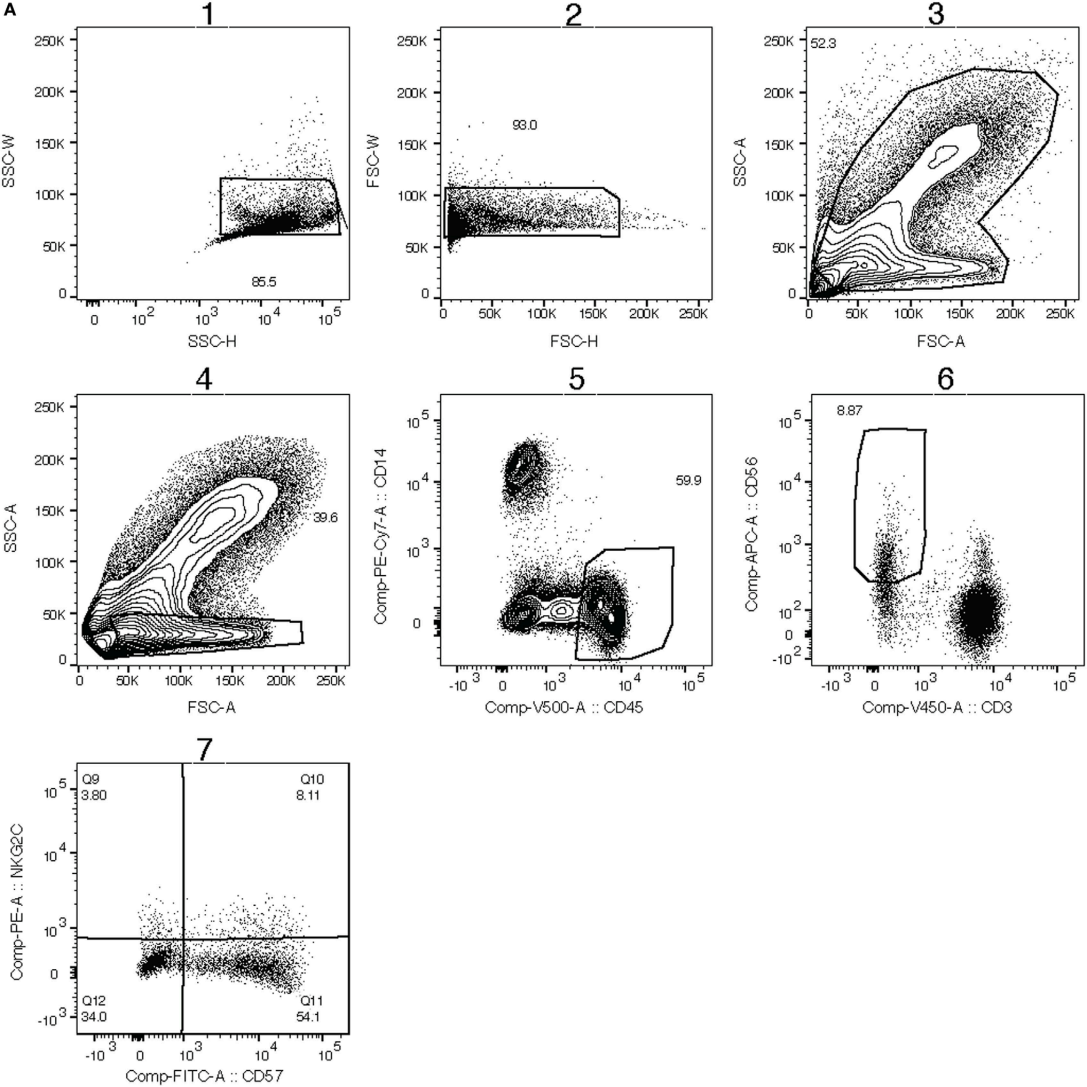

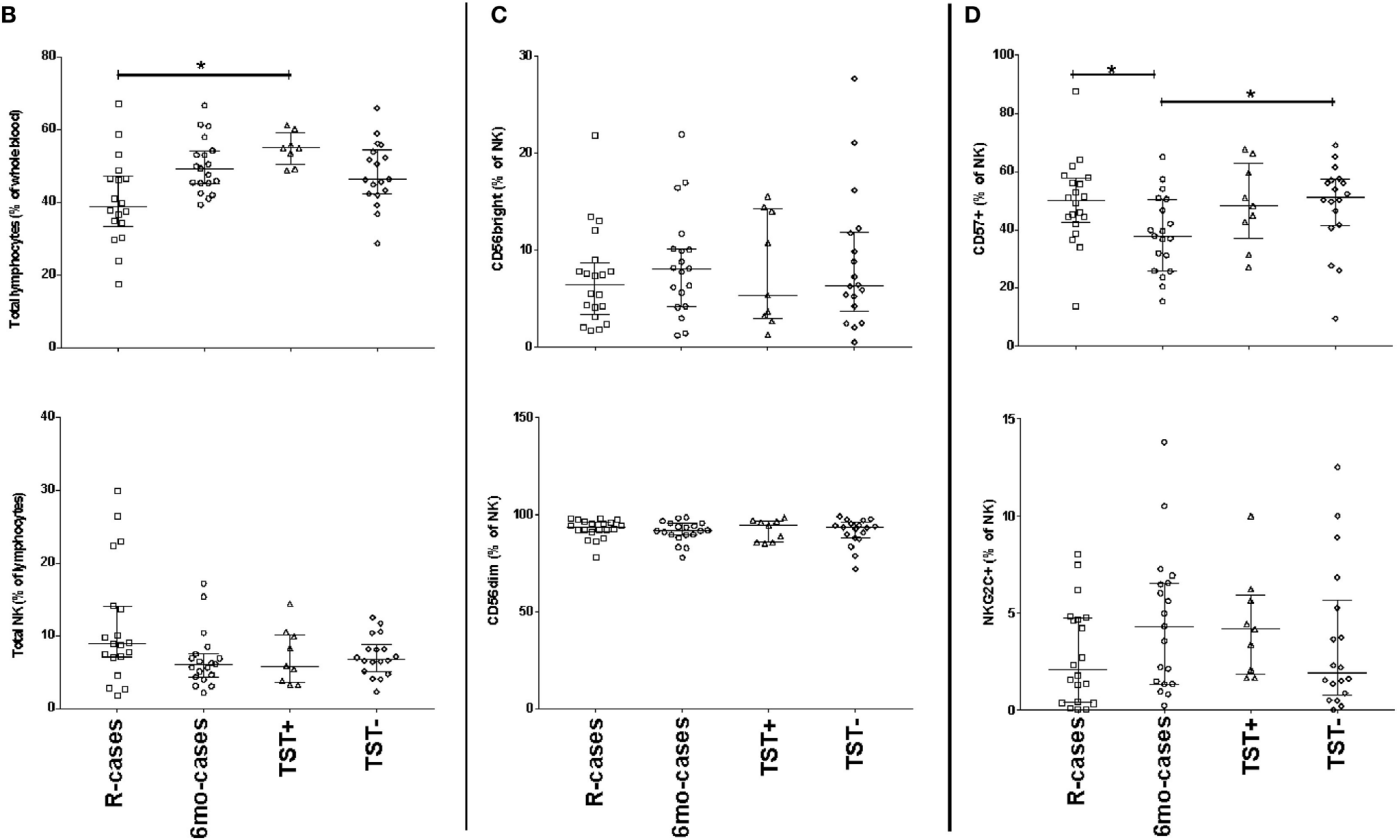
Impact of Mtb infection status on blood cell and natural killer (NK) cell subsets populations: active TB is characterized by lower lymphocyte frequencies and higher granulocyte count. Frequency (expressed as % of parent cell population) of major cell subsets analyzed by flow cytometry (unstimulated condition only). **(A)** Gating strategy with dot plots (the gating order is indicated by numbers). After doublet and debris/dead cells exclusion (plot 1), we identify the following subsets: granulocytes (plot 3, based on forward and side scatter appearance; mid-high FSC/high SSC), lymphocytes (plot 4, CD45^+^/CD14^−^), monocytes (plot 5, CD45^−^/CD14^+^), natural killer (NK) cells (plot 6, CD3^−^/CD56^dim/+^) as well as NK cells expressing CD57, NKG2C (plot 7). **(B)** Total lymphocytes (% of leukocytes), total NK (% of lymphocytes), **(C)** CD56bright (% of NK), CD56dim (% of NK), and **(D)** CD57^+^ (% of NK), and NKG2C^+^ (% of NK). Each data point represents one subject and vertical bars indicate the median ± interquartile range. Comparisons between groups (indicated below the *x*-axis of the bottom plot in each panel) were made using Kruskal–Wallis one-way ANOVA with Dunn’s test as *post hoc*; where applicable, significance is marked by an asterisk (*) and represents *p* < 0.01. The *x*-axis label represents the following four groups: (1) individuals with active TB disease = *R-cases* (open squares, *n* = 18–20), (2) 6 months post anti-TB treatment = *6mo-cases* (open circles, *n* = 18–19), (3) healthy individuals living in the same household as someone with active TB disease and has a positive tuberculin skin test (TST) result = *TST+* (open triangles, *n* = 8–9), and (4.) healthy individuals living in the same household as someone with active TB disease and has a negative TST result = *TST−* (open diamonds, *n* = 18).

Our phenotypic assessment is based on the gating strategy shown in Figure 1A. In R-cases, cell counts for granulocytes and lymphocytes were the highest and the lowest, respectively, between all participant groups (data not shown). This trend is consistent with what was observed in previous reports (59, 60). R-cases also exhibited a significant reduction in the total frequency of lymphocytes within blood leukocytes, indicating a relative enrichment of myeloid lineage (Figure 1B; top). Although there was no significant difference in NK cell frequencies between participant groups, a small increase in the total frequency of NK cells (NK defined as CD45^+^ CD3^−^ lymphocytes that expressed CD56) was observed in R-cases (Figure 1B; bottom).

CD56, the prototypical marker of NK cells, is a NCAM ligand junction type receptor and its expression is conventionally characterized as either intermediate (dim) or high (^+^). Within gated NK cells, the frequencies of CD56^+^ and CD56^dim^ subsets were compared to assess whether TB disease status impacted on NK cell functional differentiation. The frequencies of CD56^+^ or CD56^dim^ NK cells in the whole blood did not differ significantly between groups (Figure 1C). Conversely, expression of CD57, a NK cells marker associated with advanced differentiation and reduced cytokine responsiveness (61–63), was found to be significantly reduced on NK cells from 6mo-cases groups compared with that of the R-cases and contacts TST− groups (Figure 1D; top). However, CD57 expression did not differ among R-cases, TST+, and TST− groups, suggestive of a preferential expansion of less differentiated CD57^−^ cells in the 6mo-cases. The activation receptor NKG2C, which has been associated with clonal expansion and augmented effector functions upon re-exposure to the antigen (23–30), was examined to address the possible involvement of this putative innate memory-type marker in TB. The frequencies of NK cells expressing the activation receptor NKG2C were not found to differ among participant groups (Figure 1D bottom).

**Figure 2:**
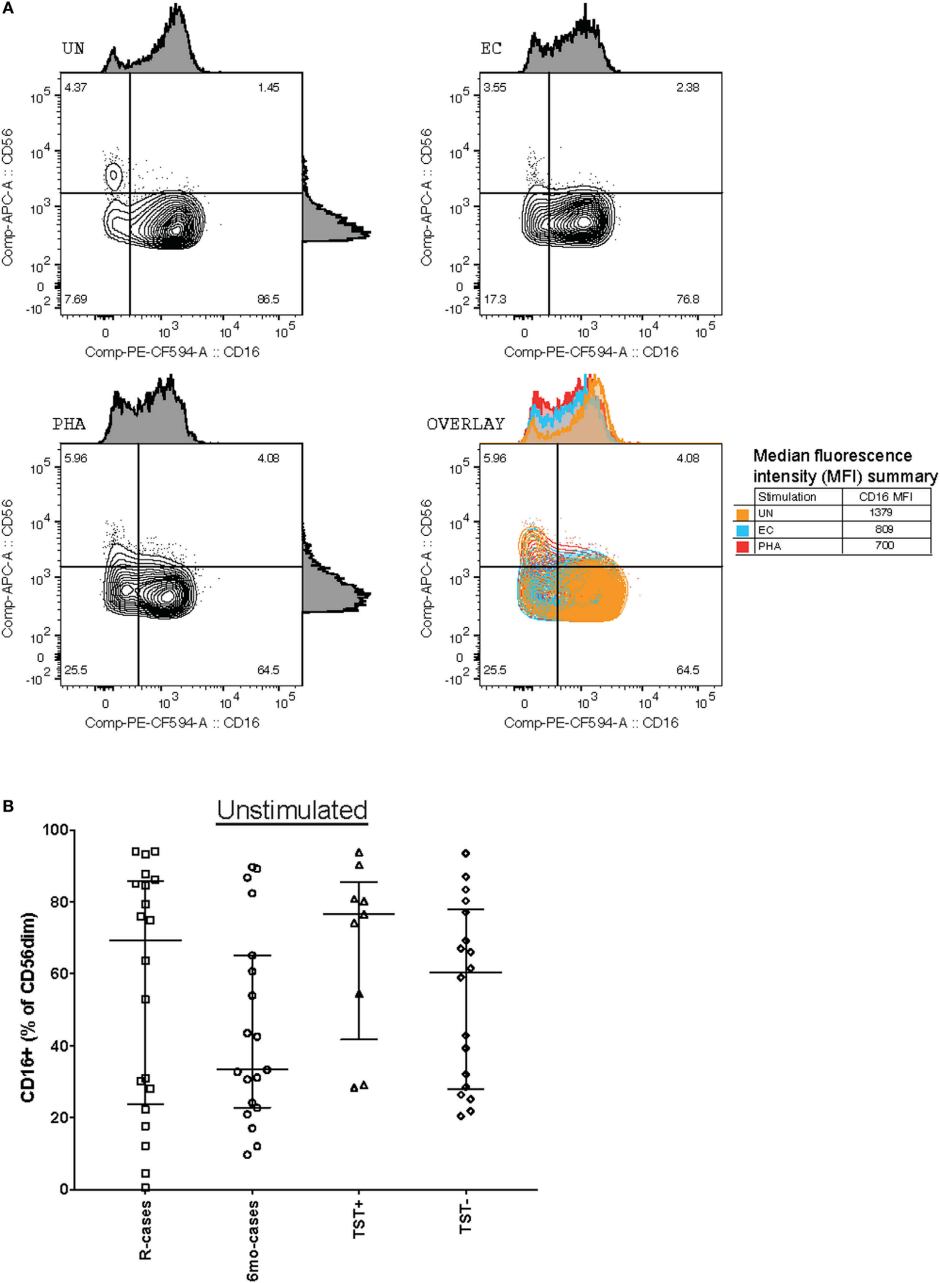

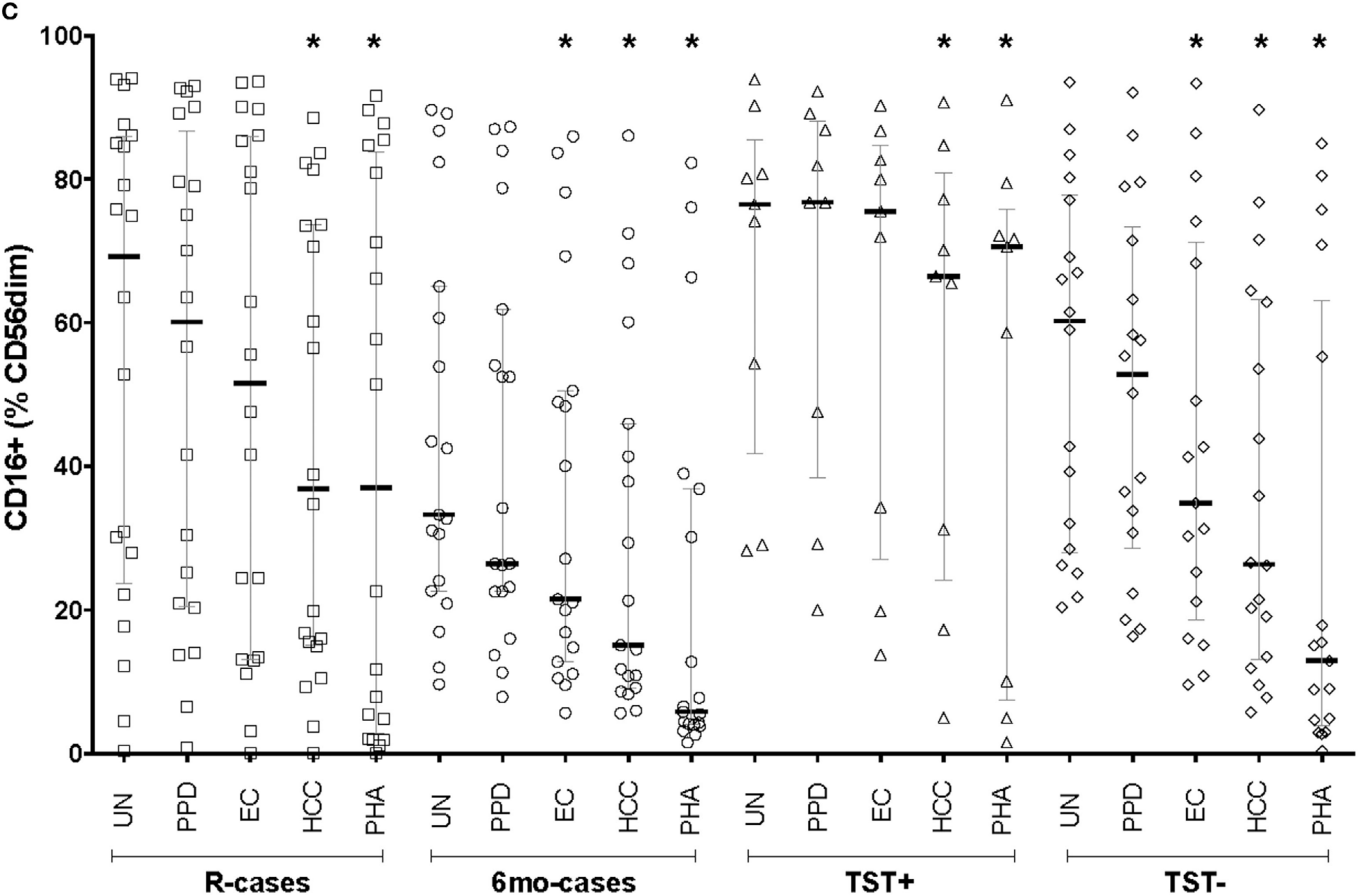
Impact of Mtb infection status and Mtb-antigen re-exposure on CD16 expression: lower baseline CD16 expression in individual who undergone 6 months anti-TB treatment compared to the pre-treatment group. The frequency of CD16^+^ cells [expressed as % CD56dim natural killer (NK) cells] analyzed by flow cytometry in the presence or absence of antigenic ligands. **(A)** Representative dot plots showing CD56 and CD16 expression on NK cells (from the CD3^−^/CD56^dim/+^ gate) from baseline (UN, top left), with exposure to EC (top right), and PHA (bottom left). CD16 median fluorescence intensity (MFI) is depicted as histograms above each plot and summarized in the overlay plot (bottom right). **(B)** Total CD16^+^ (% of CD56dim) under unstimulated condition. **(C)** Total CD16^+^ (% of CD56dim) following incubation with the following antigenic ligands: unstimulated control (UN), purified protein derivative (PPD), ESAT-6/CFP10 fusion protein (EC), high concentration of rIL-12 and rIL-18 (HCC), and phytohemagglutinin (PHA). Each data point represents one subject and vertical bars indicate the median ± interquartile range. Comparisons between groups (indicated below the *x*-axis) were made using Kruskal–Wallis one-way ANOVA with Dunn’s test as *post hoc*; where applicable, significance is marked by an asterisk (*) and represents *p* < 0.01. The effect of stimulation within each group was tested for significance using Freidman test for repeated measured with Dunn’s *post hoc* test and comparisons were performed against UN within each participant group; where applicable, significance is marked by an asterisk (*) and represents *p* < 0.01. The *x*-axis label represents the four participant groups defined in the legend of Figure 1; R-cases (*n* = 20), 6mo-cases (*n* = 19), TST+ (*n* = 9), TST− (*n* = 17–18).

Changes in the expression of CD16 cells (Fc receptor γRIIIa) could affect cytotoxicity-mediated target cell killing. Thus, the proportion of CD16^+^ NK cells between groups were compared (Figure 2). Representative dot plots showing subset frequency and CD16 median fluorescence intensity for stimulation with UN, EC, and PHA are shown in Figure 2A. The expression of CD16 was predominant in the CD56^dim^ NK subset. Note that all cell subset frequency results presented in text are expressed as the median frequency (%) of the parent cell population ± interquartile range [% (Q1–Q3)]. Participants having undergone 6 months anti-TB treatment exhibited a lowered frequency of CD16 expressing cells [33% (22–67)] compared with the other group [69% (23–85), 76% (41–85), and 60% (27–77) for R-cases, TST+, and TST−, respectively] (Figure 2B). Exposure to mycobacterial antigens and inflammatory cytokines downregulated CD16 within all groups (Figure 2C). The frequency of CD16^+^ NK cells is significantly decreased relative to the baseline (i.e., unstimulated) for all subjects stimulated with HCC and PHA, and for 6mo-cases and TST− groups stimulated with EC (Figure 2C). Sustained CD16 downregulation may play a role in modulating NK cells responses (64), therefore the extent of CD16 downregulation was compared between groups following stimulation. Of note, in the TST+ group, the magnitude of the decrease was visually lessen compared with downregulation seen in the other participant groups. A large inter-individual variation was observed, likely prohibiting any significance.

**Figure 3:**
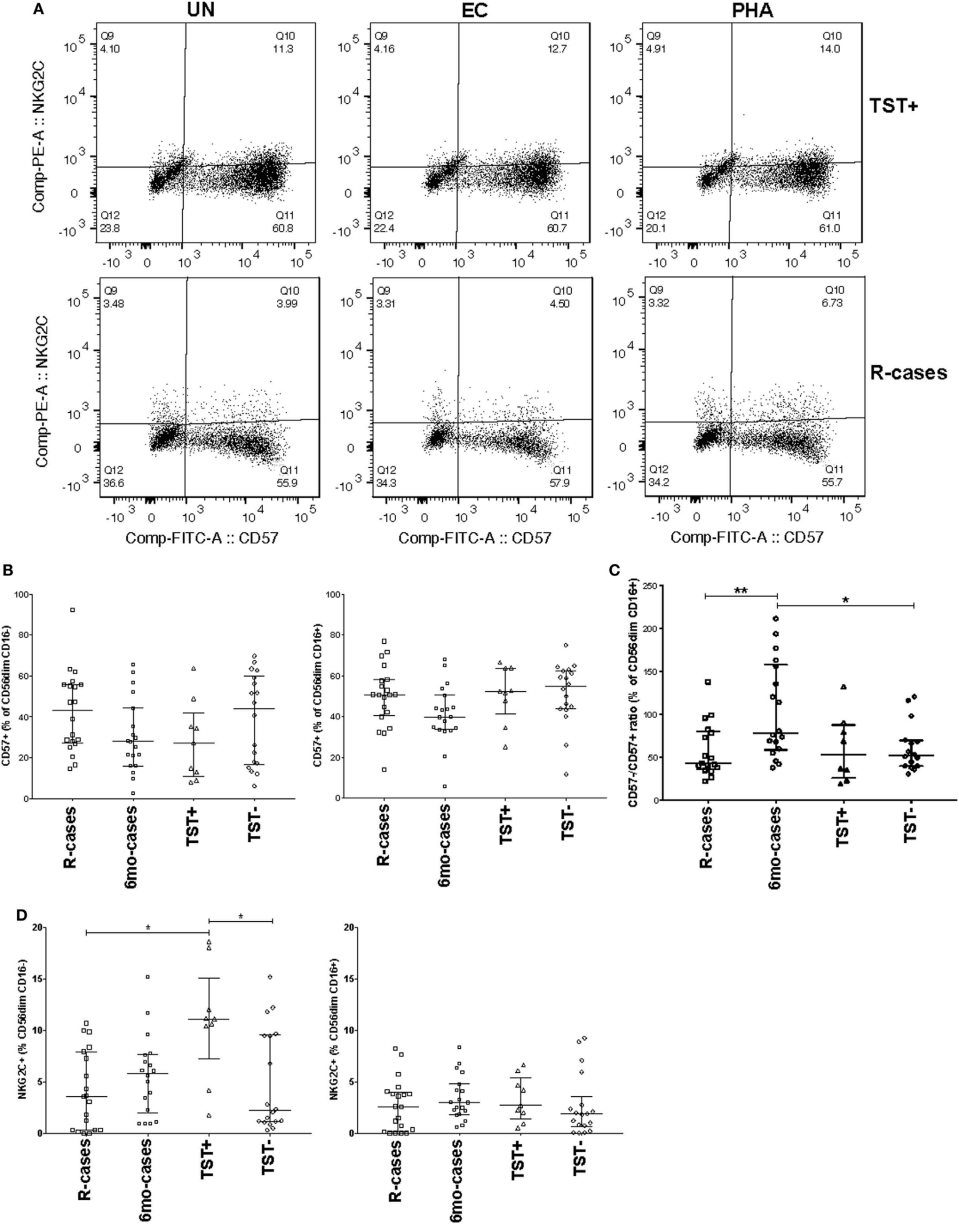
Impact of Mtb infection status on natural killer cell differentiation and maturity: (1) moderately reduced CD57 expression in individuals with prior Mtb exposure, (2) heightened expression of the activation marker NKG2C distinguishes latently infected individuals from active tuberculosis disease and healthy controls. Frequency (expressed as % of parent cell population) of major cell subsets analyzed by flow cytometry (unstimulated condition only). **(A)** Dot plots showing the expression of CD57 and NKG2C on CD56^dim^ CD16^+^ NK cells. Shown is the stimulation with UN, EC, and phytohemagglutinin (PHA) (first, second, and third columns, respectively) for individuals in the TST+ (top row) and R-cases (bottom row) groups. **(B)** Total CD57^+^ expressed as: % of CD56^dim^ CD16^−^ and% of CD56^dim^ CD16^+^ (left and right, respectively). **(C)** Ratio of CD57^−^ to CD57^+^ expressed as% of CD56^dim^ CD16^+^. **(D)** Total NKG2C^+^ expressed as: % of CD56^dim^ CD16^−^ and % of CD56^dim^ CD16^+^ (left and right, respectively). Each data point represents one subject and vertical bars indicate the median ± interquartile range. Comparisons between groups (indicated below the *x*-axis) were made using Kruskal-Wallis one-way ANOVA with Dunn’s test as *post hoc*; where applicable, significance is marked by * for *p* < 0.05 and ** for *p* < 0.01. The *x*-axis label represents the four participant groups defined in the legend of Figure 1; R-cases (*n* = 19–20), 6mo-cases (*n* = 18–19), TST+ (*n* = 9), TST− (*n* = 18).

CD57 expression was examined in the context of CD16 expression; see Figure 3A for examples of dot plots gated from the CD56^dim^ CD16^+^ NK cell subsets. The 6mo-cases group exhibited a non-significant decrease compared with R-cases and TST− (Figure 3B). However, when expressed as the CD57^−^/CD57^+^ ratio, this reduction became significant [78% (58–157), 49% (38–96), and 53% (40–84) for 6mo-cases, R-cases, and TST−, respectively] (Figure 3C). While there were no differences in the frequencies of NKG2C^+^ NK cells between groups (Figure 1D), again, significant differences were observed when examined in the context of CD16 expression. Namely, the frequency of NKG2C^+^ cells within the CD56^dim^ CD16^−^ NK subset was significantly higher in TST+ compared with that of R-cases and TST− [11% (7–15), 3.5% (0.3–7.9), and 2% (1–9.5) for TST+, R-cases, and TST−, respectively] (Figure 3D). Note that the expression of CD57 and NKG2C within CD16^±^ NK subsets remained unchanged with antigenic stimulations (data not shown).

### Functional Assessment

**Figure 4:**
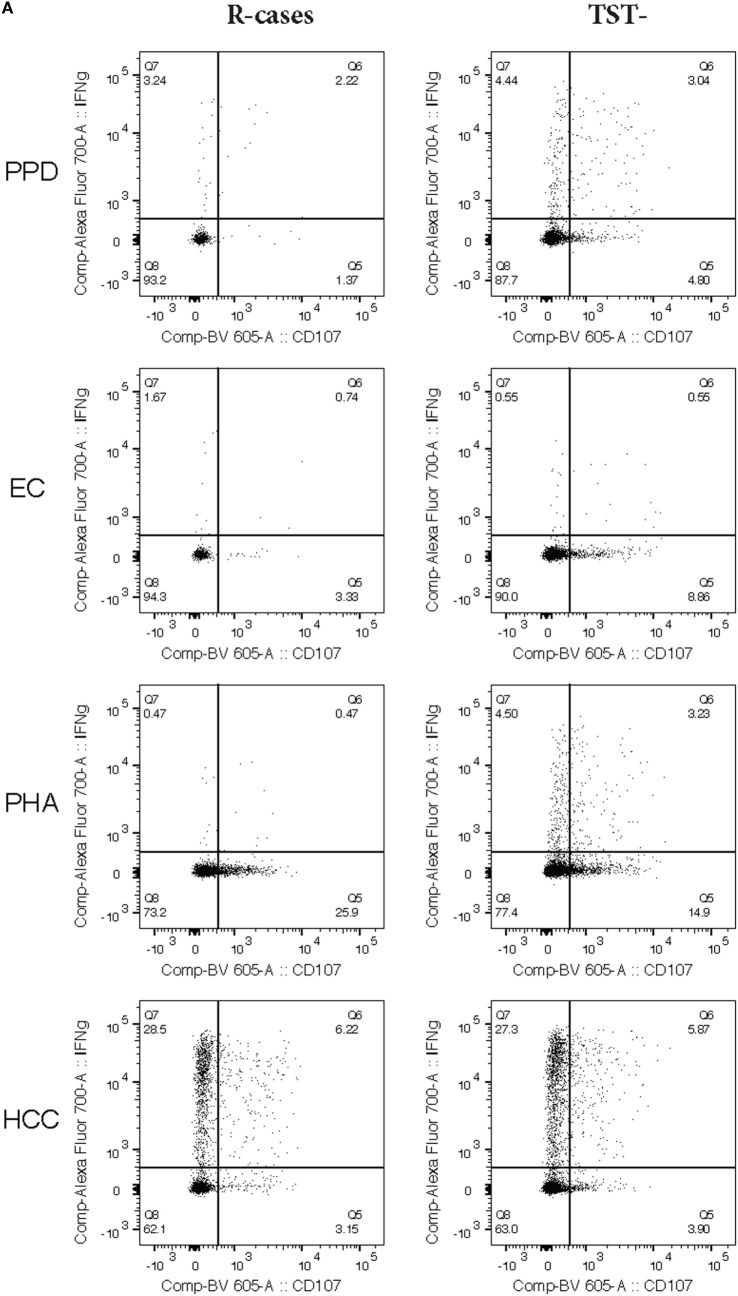

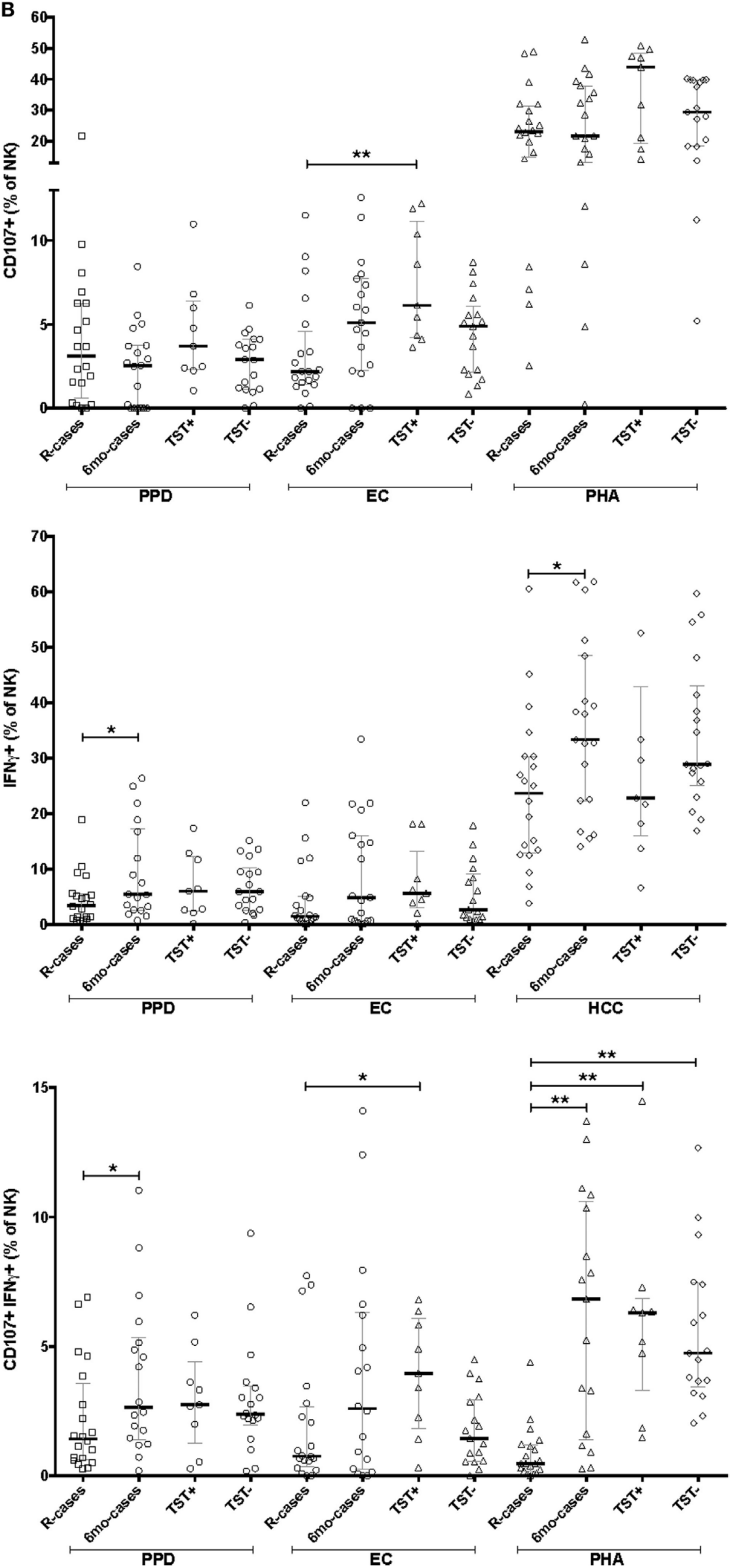
Impact of Mtb infection status and Mtb-antigen re-exposure on natural killer (NK) cell function: NK from individuals with active tuberculosis (TB) showed markedly reduced interferon gamma (IFNγ) production and moderately reduced degranulation in response to antigenic ligands. The frequency of IFNγ^+^ and CD107a^+^ cells (expressed as % NK cells) analyzed by flow cytometry in the presence or absence of antigenic ligands. **(A)** Examples of dot plots showing expression of IFNγ and CD107a by NK cells following exposure to purified protein derivative (PPD), EC, phytohemagglutinin (PHA), and HCC as indicated in the R-cases and TST− groups (left and right columns, respectively). **(B)** Total CD107a^+^ expressed following incubation with PPD, EC, and PHA (top row). Total IFNγ expressed following incubation with PPD, EC, and HCC (mid row). Total double-positive cells (i.e., IFNγ^+^CD107a^+^) following incubation with PPD, EC, and PHA (bottom row). Each data point represents one subject and vertical bars indicate the median ± interquartile range. Comparisons between groups (indicated below the *x*-axis) were made using Kruskal–Wallis one-way ANOVA with Dunn’s test as *post hoc*; where applicable, significance is marked by * for *p* < 0.05 and ** for *p* < 0.01. The *x*-axis label represents the four participant groups defined in the legend of Figure 1; R-cases (*n* = 20), 6mo-cases (*n* = 18–19), TST+ (*n* = 9), TST− (*n* = 17–18).

Natural killer cells are important mediators of innate immune response to Mtb *via* their cytotoxic activity (37, 41). Degranulation is one such important mechanism and is measured by cell-surface expression of the lysosomal marker LAMP-1/CD107a (65). The median frequencies of CD107a^+^ NK cells in response to Mtb-antigen stimulations were compared across the participant groups (see dot plots of CD107 expression for R-cases and TST− in Figure 4A). In the current study, PHA stimulation induced the highest CD107a^+^ NK cells frequencies across all groups (Figure 4B; top). After stimulation with EC, the frequency of CD107a^+^ NK cells was significantly reduced in R-cases compared with TST+ [2% (1.4–4.5) and 6% (4.2–11) for R-cases and TST+, respectively] (Figure 4B; top). Non-significant decreases were noted in R-cases compared with 6mo-cases and TST− after EC stimulation. PHA stimulation induced an increase in CD107a^+^ NK in TST+ compared with all other groups but was similar among all groups after PPD stimulation. HCC stimulation resulted in low numbers of detectable events in the CD107a^+^ gate or frequency that are similar to the unstimulated cells; thus, it was not included in the analysis.

An adequate T helper cells type 1 differentiation and IFNγ production are needed to mount a response to mycobacteria (10). NK cells are potent producers of IFNγ and signal to infected dendritic cells and macrophages necessary for mycobacteria elimination (14, 15). The median frequencies of IFNγ^+^ NK cells in response to Mtb-antigen stimulations were compared across the participant groups (see dot plots of IFNγ expression for R-cases and TST− in Figure 4A). The positive control, HCC, induced the highest IFNγ^+^ cells frequencies regardless of participant groups, while levels induced by other stimuli were similar (Figure 4B; middle). Significant increases in frequencies of IFNγ^+^ cells, compared with that of R-cases, were observed in the 6mo-cases group after stimulation with PPD [3.4% (1.1–5.5) and 5.4% (2.3–17) for R-case and 6mo-cases, respectively] and HCC [23% (12–30) and 33% (22–48) for R-cases and 6mo-cases, respectively] (Figure 4B; middle). The proportion of IFNγ^+^ NK cells was also assessed in CD56^dim^ CD16^+^, CD56^dim^ CD16^−^, and CD56^+^ NK subsets with no differences seen between the groups; however, non-significant decreases in IFNγ expression in CD56^+^ and CD56^dim^ CD16^+^ NK subsets from R-cases in response to PHA were observed (data not shown). In addition, a higher but non-significant proportion of IFNγ-producing cells in CD56^+^ and CD56^dim^ CD16^+^ NK subsets was noted in the TST+ group in response to EC (data not shown).

While the frequency of cells producing only IFNγ did not differ drastically among groups, several differences were seen in the frequency of cells with dual functional characteristic (i.e., presence of both IFNγ and CD107a) (see dot plots of CD107a and IFNγ dual expression for R-cases and TST− in Figure 4A). Frequencies were significantly lower in R-cases after PPD (Figure 4B; bottom) and HCC stimulation (data not shown) compared with that of 6mo-cases (for PPD stimulation: 1.4% (0.6–3.5) and 2.6% (1.4–5.3) for R-cases and 6mo-cases, respectively). R-cases had significantly reduced frequency of IFNγ^+^ CD107a^+^ NK cells compared to TST+ following EC stimulation [0.7% (0.3–2.6) and 3.9% (1.8–6) for R-cases and TST+, respectively] and compared to all other groups following PHA stimulation [0.4% (0.2–1.1), 6.8% (1.4–10), 6.3% (3.2–6.8), and 4.7% (3.4–7.4) for R-cases, 6mo-cases, TST+, and TST−, respectively] (Figure 4B; bottom).

**Figure 5:**
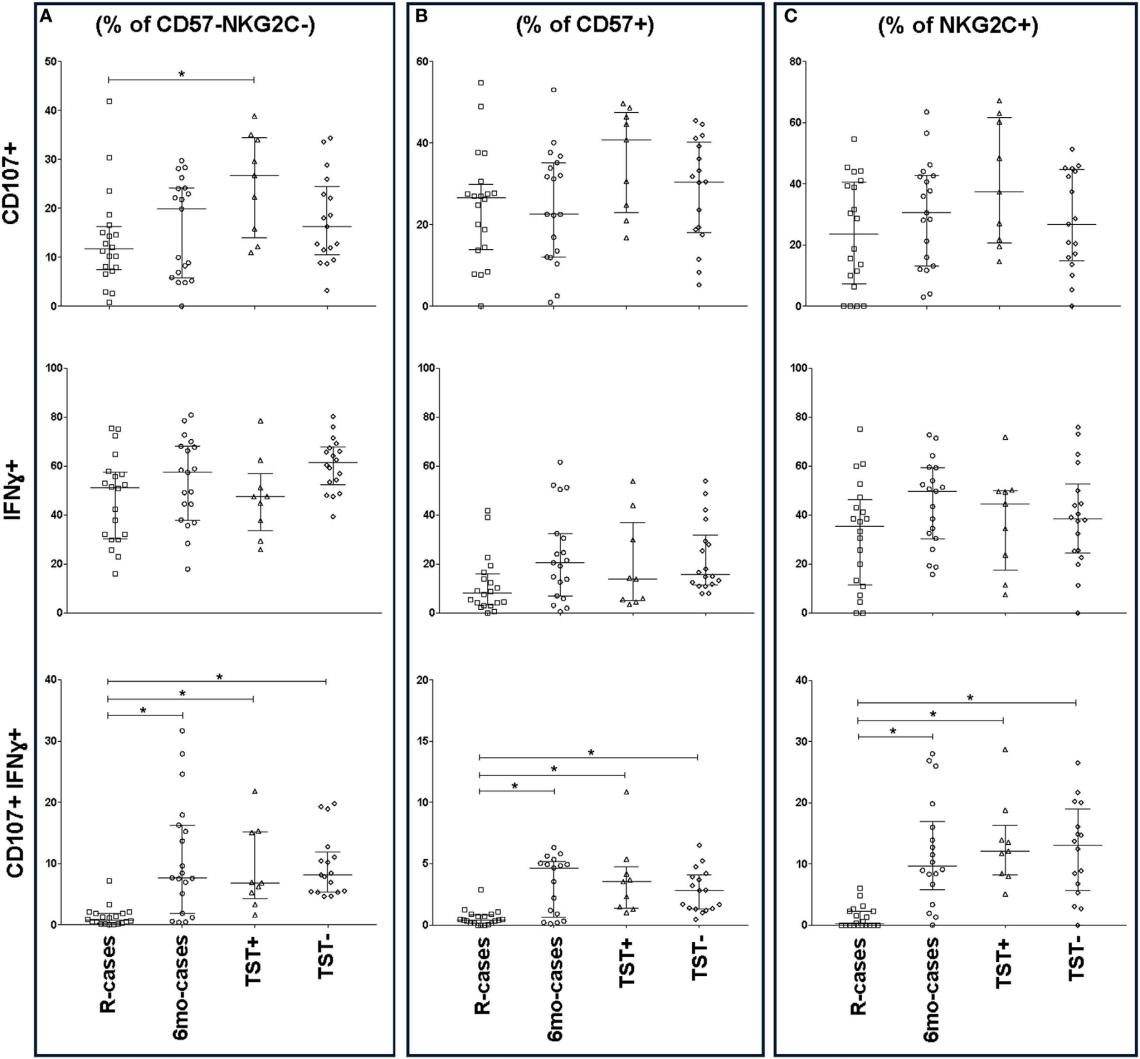
Impact of Mtb infection status on CD57/NKG2C-defined natural killer (NK) cell subset functional responses to antigenic ligands. The frequency of CD107a^+^, IFNγ^+^, and double-positive cells (top, mid, and bottom row, respectively) analyzed by flow cytometry in the presence strong antigenic ligands: phytohemagglutinin (PHA), HCC, and PHA for CD107a, interferon gamma (IFNγ), and CD107a/IFNγ, respectively. Total functional response expressed as **(A)** % of *CD57^−^ NKG2C^−^* NK cells, **(B)**% of *CD57^+^* NKG2C^−^, and **(C)** % of CD57^−^ *NKG2C^+^*. Each data point represents one subject and vertical bars indicate the median ± interquartile range. Comparisons between groups (indicated below the *x*-axis) were made using Kruskal–Wallis one-way ANOVA with Dunn’s test as *post hoc*; where applicable, significance is marked by an asterisk (*) and represents *p* < 0.01. The *x*-axis label represents the four participant groups defined in the legend of Figure 1; R-cases (*n* = 20), 6mo-cases (*n* = 18–19), TST+ (*n* = 9), TST− (*n* = 16–18).

The frequencies of IFNγ^+^ and CD107a^+^ cells were also assessed in the context of CD57 and NKG2C expression (Figure 5). Except for CD107a^+^ expression among R-cases and TST+, CD57 expression had little impact on the general CD107a expression trend seen in the CD57^−^ subset (Figures 5A,B). For IFNγ expression, there was an overall important decrease in the frequency of IFNγ^+^ cells within the CD57^+^ subset compared to CD57^−^ [e.g., 61% (52–67) within CD57^−^ versus 15% (11–31) within CD57^+^ subsets for the TST− group] (Figures 5A,B). CD57 expression had no effect on the trend and significant differences observed among groups with regard to the double-positive cells (i.e., CD107a^+^ IFNγ^+^); however, the overall frequency was half of that of the CD57^−^ subset [e.g., 8% (5.4–12) within CD57^−^ versus 2.8% (1.3–4) within CD57^+^ subsets for the TST− group].

The expression of IFNγ^+^ and CD107a^+^ in the context of NKG2C expression, are shown in Figure 5C. There was a modest increase in the overall frequency of CD107a^+^ cells within the NKG2C^+^ compared to NKG2C^−^ subsets (Figures 5A,C); however, NKG2C expression had little impact on the trend seen in the NKG2C^−^ subset (Figures 5A,C). Finally, NKG2C expression had no effect on the trends and significant differences observed between groups with regards to IFNγ^+^ and double-positive cells (Figures 5A,C).

## Discussion

Early intervention in TB pathogenesis is essential for the management of the global TB epidemic. Understanding the involvement of innate immunity during Mtb infection is critical, as the initial interaction between Mtb and immune cells provides the crucial immune cues to effector cells of the adaptive compartment (17, 66). Evidence have recently emerged in regard to the importance of NK phenotype and function during TB [reviewed in Ref. (17).]. In this study, we performed *ex vivo* whole blood stimulations which allowed comparison of NK cell subsets between individuals with different Mtb infection status.

The observed significant increase in granulocytes and decrease in lymphocytes in R-cases (compared with 6mo-cases and contacts TST−), and the increased proportion of NK cells in R-cases (compared with all other groups) were consistent with previous reports (3, 59). The lack of differences in the expression of CD56^+^ is also in agreement with a previous report (67), indicating that the pre- and post-treatment differences in IFNγ production seen in the NK cells in our study is not due to changes in the CD56^+^ population.

CD16, the Fc receptor γRIIIa, is a mediator of antibody-dependent cell-mediated cytotoxicity and is expressed by NK cells, neutrophils, monocytes, and macrophages. Antibody recognition by CD16 results in NK cells activation, release of cytolytic granules (i.e., degranulation), and target cell killing (40, 68). In the current study, participants who had undergone a 6-month anti-TB treatment (6mo-cases) exhibited a reduced frequency of CD16 expressing cells. Downregulation of CD16 following antigen recognition has been previously described (69–71) and “shedding” of CD16 have also been observed in anti-cancer therapy (72) and found to be elicited by cytokine and target cell stimulation (21). In addition, downregulation of NK CD16 expression following vaccination or re-exposure to antigens has also been associated with an enhanced antibody response that persisted for several weeks (64). Thus, the phenotype we observed in the 6mo-cases may be the end result of Mtb clearance. However, we could not exclude the contribution of the inter-individual variation in our observations; thus, further examination using paired patient whole blood, pre- and post-treatment, may provide a better assessment of the dynamic of CD16 shedding with anti-TB treatment.

A seemingly weak downregulation of CD16, especially following Mtb-specific stimulation, in latently infected individuals (i.e., TST+) was also observed. This raised the question whether CD16 expression could also be an indicator of disease progression (i.e., re-activation from latency). Distinct antibody profiles in active TB and latent TB patients were recently described by Lu et al. (73). The same study also showed that latent TB individuals have selective FcγRIII receptor binding, supporting that CD16 expression can affect Mtb clearance/control. In addition, anti-TB antibodies have been described as markers of the disease and its progression (74, 75) and evidence suggests a protective role for antibodies in TB [reviewed in Ref. (76)]. It would be interesting to further examine the potential association between anti-TB antibodies and NK cells CD16 expression and how it relates to the distinct infection status.

A marked “bi-modal” distribution for the frequency of CD16^+^ NK cells was noted within each participant group; i.e., participants regroup either near the upper range or near the lower range of frequencies. We currently do not have an explanation for this bi-modal distribution and our generalized linear model analyses confirmed that the participant characteristics (age, HIV, smear grade, or chest x-ray grade) do not have any significant effects on the results. Nonetheless, the overall trend for the group medians was consistent on an individual basis; i.e., the frequency of CD16^+^ NK cells decreased from baseline regardless of the initial baseline level. Overall, our results suggest that CD16 on its own could be an interesting marker to consider for monitoring the progression of anti-TB treatment.

Natural killer cells are important mediators of innate immune response to Mtb *via* their cytotoxic activity (37, 41). By assisting with the killing of Mtb-infected cells *via* degranulation, NK cells help control mycobacterial growth (68). In this study, the frequency of CD107a^+^ NK cells in R-cases was significantly reduced compared with the TST+ group only after EC stimulation. This suggests that NK cells from active TB patients retain the ability for cytotoxic response, but to a lesser extent when stimulated with the Mtb-specific antigen EC. NK cells are known to diversify their surface receptors *via* the adaptation and response to pathogens, environmental cues (77), and specific ligands (78). Such diversification results in the expression of various patterns of activating and co-stimulatory receptors (77). It is likely that changes in the NK cell repertoire during the progression of the disease (i.e., from latent to active) resulted in the observed differential response to EC. Thus, differences in degranulation and CD16 expression between R-cases and TST+ could be potential markers for differentiating active disease from latency.

Interferon gamma is a well-known critical factor in the protection against Mtb infection (9) and many diagnostic tools of TB are based on IFNγ production upon stimulation with Mtb-specific antigens [reviewed in Ref. (3).]. Response to mycobacteria and other intracellular pathogen requires an adequate T helper cells type 1 (Th1) differentiation and IFNγ production. However, there are important discrepancies in the levels of IFNγ produced by T cells in response to *ex vivo* stimulation, especially in regard to treatment response (3): IFNγ production in TB cases after treatment has been shown to be higher (79, 80), similar (81, 82), or lower (82, 83) compared with level before treatment. For the most part, the discrepancies have not been attributed to any specific parameter and remain unexplained (84) and call for the potential role of innate cells as early mediators of protective immunity against Mtb infection.

The delayed activation of effector T cells is one of the major bottlenecks to Mtb clearance and could be due to an impaired signaling to and from innate cells (4). NK cells are potent producers of IFNγ and provide signal to infected dendritic cells and macrophages to assist with mycobacteria elimination. In this study, IFNγ^+^ NK cells in R-cases were significantly reduced, with the greatest difference from that of the 6mo-cases group, indicating that important functional changes had occurred in NK cells following Mtb clearance. Enhancement of IFNγ responses in the 6mo-cases could result from infection-induced pre-activation of NK cells, cytokine-induced memory-like NK cells generation (85), and/or help from CD4^+^ T cells.

In regards to polyfunctionality, Vahlne et al. have described the phenotypic differences between IFNγ^+^, IFNγ^+^ CD107a^+^, and CD107a^+^ NK cells and showed that ligands and cell maturity can affect the frequency of each of the phenotypes (86). Immature NK cells mainly express CD107a, thus, are more cytotoxic. NK cells of intermediate maturity secrete IFNγ and express CD107a, while mature NK cells mainly secrete IFNγ (86). Our results showed that R-cases had the most reduced frequency of IFNγ^+^ and IFNγ^+^ CD107a^+^ NK cells, but were able to retain some CD107a^+^ NK cells at levels similar to that of TST− and 6mo-cases groups. This suggests that NK cell in R-cases are mostly immature and cytotoxic focused. However, additional studies are needed to determine whether the maturation of NK cells, the production of IFNγ, or the accumulation of IFNγ is affected specifically in R-cases. Similarly, whether any of these processes are the instigating factors for the establishment of the disease or consequences of active TB disease remains to be investigated.

The surface marker CD57 can be found on ~10–20% of lymphocytes, as well as in some epithelial, neural, and chromaffin cells (87). Among lymphocytes, CD57 positive cells are typically T cells or NK cells and are most commonly found in the germinal centers of the lymph nodes, tonsils, and spleen (87). NK cells expressing CD57 are more differentiated (61, 88). They are associated with CD16 expression, less responsive to cytokine signaling (62), and produce lower IFNγ in response to antigenic stimulation (61). Those characteristics of CD57^+^ NK cells were also observed in the whole blood in this study; i.e., the majority of IFNγ^+^ NK cells were CD57^−^ while CD107a^+^ NK cells were CD57^+^. Significantly less CD57^+^ NK cells were also found in 6mo-cases compared with R-cases and TST−. Together, these observations suggest the replenishment of the less differentiated, cytokine-responsive, and IFNγ producing NK cell subset following Mtb clearance; i.e., a reversal in the repertoire of NK cell subsets. Interestingly, HCMV infection has also been shown to drive preferential expansion of CD57^+^ and NKG2C^+^ NK population, i.e., toward a less cytokine-responsive phenotype (63). Thus, the differentiation of NK cell could also be a potential marker of TB treatment progression/outcome.

Environmental factors, such as viruses, can have profound impact on the functionality and adaptation of innate cells, a feature previously ascribed to adaptive immune cells only (89). Such environmental impact, such as modulation of innate immune responses and susceptibility to infection, is apparent in the study by Maertzdorf et al., where NK-associated genes were significantly downregulated in active TB patients compared with those with latent TB infection (90). These observations suggest that NK cells activity is a major controlling factor of Mtb infection and that there are significant changes in the NK cell repertoire with infection status.

One particular NK-specific surface marker, NKG2C, highlights the adaptive nature of NK cells during chronic diseases; i.e., the specific expansion of a NK cell subset upon re-exposure with specific antigen. NKG2C is an activating receptor which binds to HLA-E in a ligand-dependent affinity (91) and is expressed mainly on CD56^dim^ NK cells (92, 93). NK cells expressing NKG2C are clonally expanded during viral infection (24, 26, 30) and have enhanced effector functions [reviewed in Ref. (89)]. Our phenotypic results in the whole blood echoed the ideas put forth by Rolle and Brodin (89) and the findings of Maertzdorf et al. (90)—the proportion of NKG2C^+^ CD56^dim^ CD16^−^ NK cells was significantly elevated in TST+ compared with levels in R-cases and TST−. The increased frequency of imprinted NK cell memory in latently infected individuals, which could be the result of repeated Mtb exposure, could play an important role in the re-activation of TB. Similar expansion of NKG2C^+^ NK cell subset has also been observed in patients with reactivated CMV (26, 63). Furthermore, the lack of differences between pre and post TB treatment observed in this study is consistent with the unique aspect of NKG2C^+^ NK cells to have the potential to distinguish latent versus active TB.

### Limitations

A larger sample size could reduce the large inter-individual variation observed in the frequencies of the various NK subsets as well as improving the power of the statistical comparisons, which was quite stringent with the inclusion of multiple comparisons correction. However, such inter-individual variability could also be the results of surface receptor diversification; e.g., CD16 has one of the largest expression diversity among donors even though it is a minimally polymorphic receptor (77). In addition, Fauriat et al. showed that specific ligands dictate the quality and timing of NK cells cytokine production. Therefore, the magnitudes of the specific immune responses from NK cells are likely graded depending on repertoire of activating receptors expressed and utilized (22, 78). Of note, NKG2C genotype may contribute/modulate the expansion of NKG2C^+^ NK cells (31–33, 36, 94); however, in our study, we did not perform individual genotyping of the NKG2C gene. The Mtb strains are another important variable as strains have been shown to be associated with variation in IFNγ production in response to Mtb antigens (95); unfortunately, data on the strains were not available for the current study. A gender bias was observed in our study which is representative of the recruitment at our study site and can likely be explained, in parts, by social factors (96, 97); consequently, correcting for gender was not suitable. Lastly, longitudinal whole blood samples from active TB patients, i.e., pre- and post-treatment (paired), would allow for a more direct assessment of treatment effect.

## Conclusion

We have obtained new insights into the role of NK cells during Mtb pathogenesis in a physiologically relevant matrix through characterizing the changes in NK cells phenotype and function in response to Mtb antigens. Using multicolor flow cytometry, we found that NK cells of active TB patients produced significantly reduced IFNγ and degranulation in response to Mtb-specific antigens. We also noted that TB treatment is associated with a lowered frequency of CD57^+^ NK cells, suggesting an alteration of NK repertoire in favoring cells to become more responsive to cytokines and activation. There were distinctive differences between R-cases and TST+ in respect to CD107a and CD16 expressing cells, providing future opportunities to distinguish active versus latent disease using those markers. In addition, TST+ individuals exhibited a higher frequency of NKG2C^+^ NK cells, suggesting that NK cells have gained, from previous exposure to Mtb, a primed or licensed NK cell population. These new insights gained from this profiling of host immunity characteristic of NK cells hold promise as a tool to delineate the immunological mechanism involve at different stage of Mtb infection.

## Ethics Statement

This study was carried out in accordance with the recommendations of the Medical Research Council (MRC) Unit The Gambia (MRCG) with written informed consent from all subjects. All subjects gave written informed consent in accordance with the Declaration of Helsinki. The protocol was approved by the Gambian Government-Joint Ethics Committee.

## Author Contributions

M. Garand designed and conducted the experiments as well as interpreted the data, analyzed all results, and wrote the manuscript. OO and SD identified suitable donors, and collected clinical data and blood samples. JS provided inputs on design. JS and M. Goodier edited the manuscript and provided comments. BK and JS provided funding.

## Conflict of Interest Statement

The authors declare that the research was conducted in the absence of any commercial or financial relationships that could be construed as a potential conflict of interest.

## Supplementary Material

Figure S1 Fluorochrome gating controls (all-minus-one). Precise gating for key cell populations was controlled using mixes of antibody–fluorochrome pairs in which one specific antibody–fluorochrome pair is missing; these mixes are called all-minus-(specific target name). As indicated above each dot plot, fluorochromes tagged to anti-CD45, CD56, CD16, IFNγ, CD107a, CD57, and NKG2C were omitted one-by-one from the antibody mix and gates or quadrants were placed in order to have frequency <0.5% detected for that missing pair.
