# Supplementary figures and images for: Functional and Phenotypic Changes of Natural Killer Cells in Whole Blood during Mycobacterium tuberculosis Infection and Disease
Source: Front Immunol. 2018 Feb 19;9:257. doi: 10.3389/fimmu.2018.00257 (PMC5827559; doi:10.3389/fimmu.2018.00257)

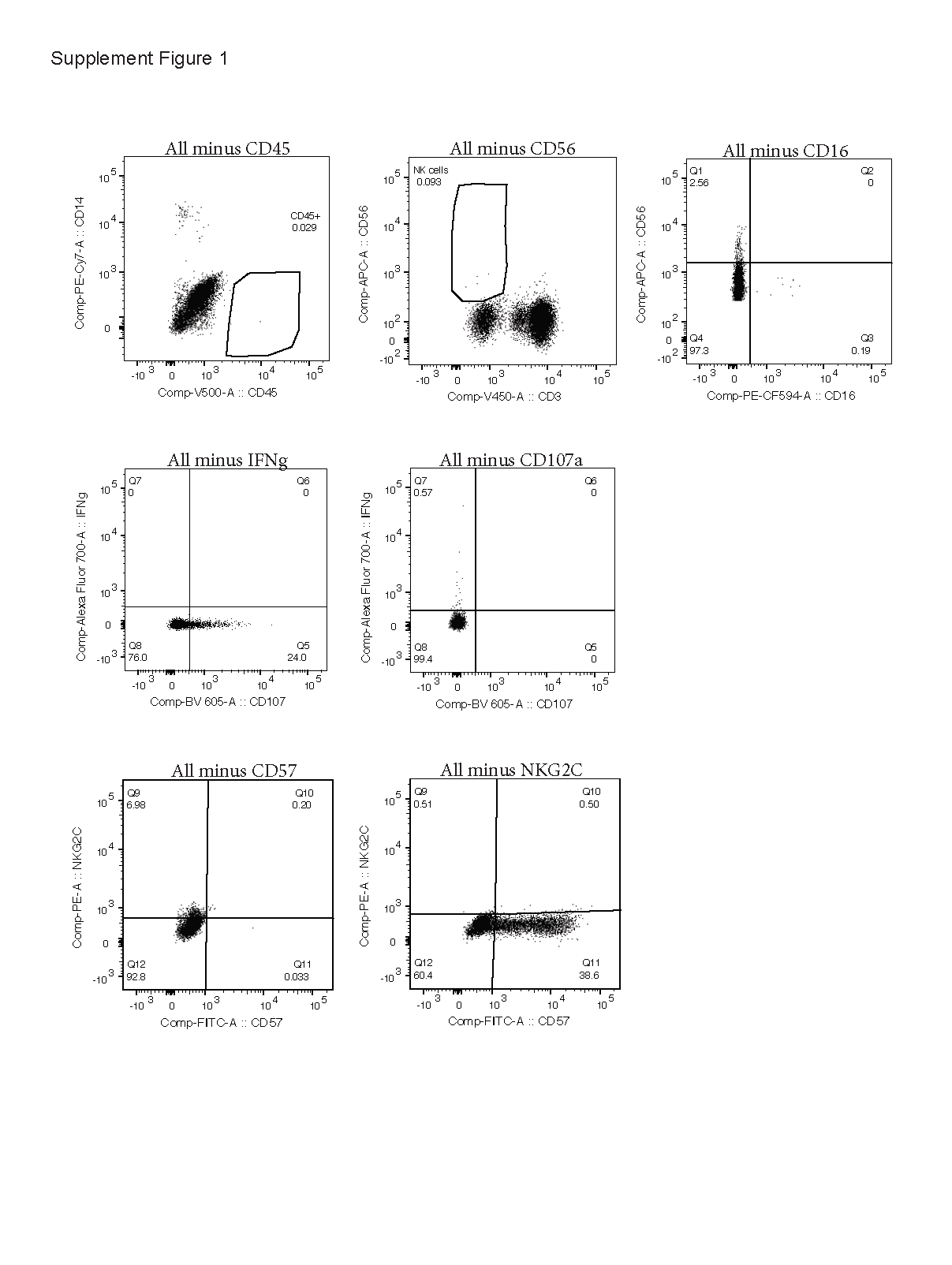

Supplement: Figure S1 — Fluorochrome gating controls (all-minus-one). Precise gating for key cell populations was controlled using mixes of antibody–fluorochrome pairs in which one specific antibody–fluorochrome pair is missing; these mixes are called all-minus-(specific target name). As indicated above each dot plot, fluorochromes tagged to anti-CD45, CD56, CD16, IFNγ, CD107a, CD57, and NKG2C were omitted one-by-one from the antibody mix and gates or quadrants were placed in order to have frequency <0.5% detected for that missing pair. [file Image_1.tiff]
